# Complete Genome Sequence of the Methicillin-Resistant Staphylococcus aureus Strain JMUB3031, Isolated from a Patient with Fatal Community-Acquired Pneumonia

**DOI:** 10.1128/MRA.01652-18

**Published:** 2019-01-24

**Authors:** Bintao Cui, Shinya Watanabe, Yusuke Sato’o, Fumiya Nihashi, Yoshifumi Aiba, Kotaro Kiga, Teppei Sasahara, Xin-Ee Tan, Moriyuki Kawauchi, Tanit Boonsiri, Kanate Thitiananpakorn, Yusuke Taki, Feng-Yu Li, Shiro Imokawa, Longzhu Cui

**Affiliations:** a Division of Bacteriology, Department of Infection and Immunity, School of Medicine, Jichi Medical University, Shimotsuke, Japan; b Department of Respiratory Medicine, Iwata City Hospital, Iwata, Japan; University of Rochester School of Medicine and Dentistry

## Abstract

Severe community-acquired pneumonia (CAP) caused by methicillin-resistant Staphylococcus aureus (MRSA) is relatively rare and is usually associated with rapid progression to death. Here, we report the complete genome sequence of the MRSA strain JMUB3031, which was isolated from a patient with fatal CAP.

## ANNOUNCEMENT

Community-acquired pneumonia (CAP) caused by methicillin-resistant Staphylococcus aureus (MRSA) is defined as a lung inflammation in which MRSA is cultured from the blood or sputum of an outpatient or an inpatient within less than 48 h of hospital admission. Although globally rare, this disease can result in significant morbidity and mortality (1, 2).

The MRSA strain JMUB3031 was isolated from a 46-year-old man who was a diabetic smoker. He presented with influenza-like symptoms, including fever, headache, low appetite, and dyspnea. His hospital examination revealed high fever (39°C) and a respiratory rate of 35 breaths/min. Unfortunately, the patient died 9 h after hospital admission. Bacterial cultures revealed MRSA in his blood, sputum, and bronchial lavage samples obtained on the day of hospital admission and in lung autopsy samples obtained after his death, from which no antecedent influenza infection was detected. The complete genome sequence of strain JMUB3031 was determined to further investigate the molecular determinants that render it virulent.

Two whole-genome sequencing approaches were used, (i) long-read sequencing (PacBio RS II; Pacific Biosciences of California, Inc., USA) and (ii) mate pair sequencing using the Illumina MiSeq platform (Illumina, Inc.). Genomic DNA was extracted using the NucleoBond AXG kit (TaKaRa Bio, Inc., Japan) for long-read sequencing or the phenol-chloroform method for mate pair sequencing (3). The genome was sequenced using a PacBio RS II system on a single-molecule real-time (SMRT) cell, which generated 112,990 filtered reads with a mean length of 12,302 bp. *De novo* assembly was performed using the Hierarchical Genome Assembly Process (HGAP version 3), which produced two assembled sequences (2,863,867 bp and 33,992 bp), with a coverage of 231×. Mate pair sequencing was carried out as previously described (4) and generated 4,208,676 paired-end reads. After quality trimming using the FASTQ toolkit (version 2.0.0) with a quality level of 30, a total of 2,250,406 high-quality reads were assembled into contigs and scaffolds with the Velvet *de novo* assembler (version 1.2.10) algorithm. The resulting assembly comprised 47 scaffolds, of which 45 were short sequences. After evaluation, a 2,823,658-bp chromosome sequence and a 21,407-bp plasmid sequence were identified. Thirty-seven gaps of the chromosomal scaffold were filled by PCR, followed by Sanger sequencing using an ABI3130xl genetic analyzer (Applied Biosystems, Carlsbad, CA). Mismatches between the PacBio and mate pair sequences were then confirmed using Sanger sequencing. To assess the plasmid sequence, comparison of experimental restriction fragment length polymorphism (RFLP) profiles using isolated plasmid DNA with *in silico* restriction prediction was performed with NEBcutter 2.0 (http://www.labtools.us/nebcutter-v2-0/), affirming that the mate pair plasmid sequence was reliable. Gene extraction and annotation were performed with the Microbial Genome Annotation Pipeline (http://www.migap.org).

S. aureus JMUB3031 harbors a circular chromosome of 2,864,283 bp (G+C content, 32.75%) and a plasmid of 21,326 bp. A total of 2,657 coding sequences, 50 tRNA genes, and 8 rRNA genes were identified on the chromosomal genome.

JMUB3031 belongs to sequence type 1 (ST1) and clonal complex 1 (CC1), which were identified by the Multi Locus Sequence Typing (MLST) website (http://www.mlst.net/) (5), and to SCC*mec* type IVc (6). Three prophages and virulence genes likely involved in severe pneumoniae were identified. The Panton-Valentine leukocidin (PVL) genes *lukS-PV* and *lukF-PV*, adjacent to the enterotoxin gene *sea,* are located on ϕSa3, representing an architecture different from that of MW2, the most related ST1 CAP-MRSA strain, in which the PVL genes and *sea* are separately located on ϕSa2 and ϕSa3 of MW2 (7, 8). Other enterotoxin genes, such as *seh*, *seq*, *selx*, and the gene encoding α-hemolysin (Hla), were identified on its chromosome, which may also be associated with MRSA CAP (9–11).

### Data availability.

The genome sequence was deposited in DDBJ/GenBank under the accession numbers AP018923 (chromosome) and AP018924 (plasmid), and the raw sequence data were deposited in DDBJ/Sequence Read Archive (DRA007604).
